# Antimicrobial resistance trends during COVID-19 in male patients: a descriptive analysis

**DOI:** 10.25122/jml-2026-0034

**Published:** 2026-03

**Authors:** Răzvan-Ionuț Popescu, Răzvan-Cosmin Petca, Cristian Mareș, Aida Petca, Osamah Shehab Ahmed, Leonard Ostafi, Viorel Jinga

**Affiliations:** 1 Carol Davila University of Medicine and Pharmacy, Bucharest, Romania; 2 Department of Urology, Prof. Dr. Th. Burghele Clinical Hospital, Bucharest, Romania; 3 Department of Urology, Saint John Clinical Emergency Hospital, Bucharest, Romania; 4 Department of Obstetrics and Gynecology, CF2 Clinical Hospital, Bucharest, Romania

**Keywords:** urinary tract infection, male patients, antimicrobial resistance, Gram-negative, Gram-positive

## Abstract

Urinary tract infections (UTIs) in male patients represent a distinct and underexplored clinical entity. The COVID-19 pandemic has further influenced infection patterns and antimicrobial use, potentially accelerating resistance trends. This study aimed to evaluate the prevalence of uropathogens and their resistance patterns in a large cohort of Romanian men during the full pandemic. A retrospective descriptive study was conducted at a tertiary academic urology center in Bucharest, Romania, including male patients evaluated during the COVID-19 pandemic (September 2021–February 2022). Male patients with positive urine cultures (≥10^5^ CFU/mL) and a single identified pathogen were included, while duplicate and polymicrobial samples were excluded. 733 male patients were included, with Gram-negative predominating (78.99%) over Gram-positive (21.01%). *E. coli* (32,74%) and *Klebsiella* (27.15%) were the most frequent isolates. UTIs were markedly more prevalent in patients >55 years. Gram-negative uropathogens showed high resistance to commonly used antibiotics, particularly Amoxicillin-Clavulanic acid (43.98%) and Levofloxacin (39.16%). *E. coli* demonstrated elevated resistance to Levofloxacin (36.52%) and Amoxicillin-Clavulanic acid (35.78%), while *Klebsiella* exhibited the most concerning profile, exceeding 50% resistance to Amoxicillin-Clavulanic acid (57.29%) and Nitrofurantoin (56.96%), with additional carbapenem resistance (Imipenem 13.59%, Meropenem 12.9%). Gram-positive isolates displayed relatively stable resistance patterns, with *Enterococcus* showing high resistance to Levofloxacin (56.7%) and lower resistance to Penicillin (19.67%). The male population showed higher resistance rates to several antibiotics, possibly due to the more complicated nature of their infections. These data highlight the need for tailored therapeutic approaches based on gender-specific resistance patterns.

## Introduction

Urinary tract infections (UTIs) pose a significant public health concern, carrying substantial socio-economic ramifications that impact a considerable portion of the population. The high prevalence of this pathology is impacting a global population of more than 150 million individuals every year [1]. Recent studies estimate that there are over 10 million outpatient visits in the United States and around 2 million emergency room visits yearly [2,3].

The issue of antimicrobial resistance poses a significant and escalating global barrier to providing medical treatment. Consequently, there has been a notable escalation in expenses related to patient care, a surge in the duration of hospital admissions, and a corresponding rise in mortality rates. Research has revealed that the most common infections encountered in clinical settings demonstrate notable resistance to traditional antibiotic therapies. Furthermore, reports indicate that numerous organisms exhibit multidrug resistance [3].

Due to the higher rate of incidence in comparison to males, the vast majority of research on urinary infections has been conducted in female patients. Because of this, guidelines on UTIs accept studies on female patients as reference, even though there are significant anatomical differences between the genitourinary systems of men and women. These are the primary causes of morbidity that affect people of all ages and involve both sexes. People of both sexes can be affected. These infections are characterized by a high multiplication of a range of pathogens along the urinary system, which results in an inflammatory condition that leads to malfunction of the kidneys or other components of the urinary apparatus. Additionally, these infections are associated with a higher risk of developing urinary tract cancer [4,5].

UTIs have a greater impact on women due to their increased susceptibility over the course of their lives. However, as men age, they experience certain anatomical and functional changes that impact the process of proper voiding [6]. These changes mostly contribute to the heightened susceptibility to infection among the senior population. The incidence of more complex infections tends to be higher in elderly males due to prostate enlargement, which contributes to bladder obstruction, urine stasis, and the manifestation of the disease. Men with urinary tract anomalies are susceptible to recurrent infections caused by highly aggressive microorganisms. The presence of resistant bacteria that poor treatments cannot eliminate can also contribute to the development of these infections. Several obstructive pathologies, such as benign prostatic hyperplasia, urethral stricture, and bladder neck obstruction are the primary etiological factors associated with urinary tract infections in males [7].

UTIs can be categorized into two main types: healthcare-associated urinary tract infections (HAUTIs) and community-associated UTIs (CAUTIs). Catheter-associated urinary tract infection (CAUTI) is frequently observed in female patients with certain risk factors, including advanced age, a previous history of UTI, sexual activity, and diabetes mellitus. On the other hand, healthcare-associated urinary tract infection (HAUTI) is closely associated with medical treatments that occur within a hospital setting. In this context, indwelling urinary catheters are regarded as the primary risk factor [8].

The most prevalent uropathogens associated with urinary tract infections (UTIs) are *Escherichia coli* (*E. coli*), which accounts for approximately 33% of cases, as well as *Enterococcus faecalis, Klebsiella pneumoniae*, and *Proteus mirabilis*. The specific frequencies of these pathogens may vary depending on the type of UTI, whether it is community-acquired (CAUTI) or healthcare-associated (HAUTI) [9].

The incidence of antibiotic resistance in both catheter-associated urinary tract infections and hospital-acquired urinary tract infections are highly dependent on the geographical location [8]. The high occurrence of bacterial causation in UTIs leads to a significant utilization of broad-spectrum antibiotics, resulting in elevated levels of uropathogens that are resistant to these medications [10]. The increased prevalence of multidrug resistance (MDR) in UTIs can be attributed to the widespread use of empirical antibiotics and the transmission of antibiotic-resistant genes and other resistance determinants carried by mobile genetic elements. This has resulted in the challenging task of treating UTIs, even at the community level [11].

Antibiotics were one of the most important discoveries in modern medicine, and they have been used to treat a wide range of infections with clear benefits. Resistant uropathogenic bacteria are becoming more common because of overuse and abuse, which is also a very serious threat to public health. The European Association of Urology (EAU) guidelines say that all antibiotics should be used carefully, and they suggest that Antibiotic Stewardship be a key part of everyday clinical practice. It also suggests changing the antibiotic use rules based on the rates of resistance and sensitivity found in each area. This will help slow down the rise in resistance and make antibiotics more effective [12].

In a recent report, the European Centre for Disease Prevention and Control identified Romania, as one of the European Union nations with the lowest rates of resistance to antibiotic agents. However, it is noteworthy that Romania also has one of the highest consumption rates [13,14]. The aforementioned observation should be regarded as a cautionary indication for the medical community to advance their research in order to enhance the conscientiousness of drug administration.

One potential strategy to address the growing issue of antibiotic resistance is the customization of antibiotic usage policies based on the specific rates of resistance and sensitivity seen in the local population. This approach has the potential to not only slow down the progression of resistance, but also enhance the effectiveness of empirically prescribed antibiotics [12]. Limited recent data on local resistance patterns in the Romanian territory are available for the moment [3,15-17].

Most studies investigating the prevalence and antimicrobial resistance reflect data obtained from the female population. According to the guidelines in force, the treatment indications are made based on the data obtained from the female sex. Considering that both the associated pathology and the age at which these infections develop among the male population differ, it is expected that the prevalence and resistance data of uropathogens will also show changes. It is also very likely that there are differences in terms of the loco-regional space and the investigated periods. In Romania, there are very few studies that aim to evaluate this topic. This article aims to evaluate the prevalence of uropathogens and resistance rates to the common antibiotics used in current practice on a significant group of male patients registered in Bucharest, at “Prof. Dr. Th. Burghele” Clinical Hospital, one of the largest urology centers in Romania.

## Material and Methods

### Study design and population

The present research is a descriptive retrospective study including patients from a highly representative urology center and also an academic institution in Bucharest, Romania: “Prof. Dr. Th. Burghele” Clinical Hospital (BCH). The obtained data covered a 6-month period, from September 1, 2021, to February 28, 2022, during the full COVID-19 pandemic, and included only male patients.

Following the analysis of 16,317 cases carried out in the mentioned period, 3,789 cases with positive urinalysis were identified. Of these, 733 samples were identified as positive samples in male patients. Cases with the same urine culture results at two different analyses were excluded.

In all cases, the designated patients were hospitalized or received outpatient care. Therefore, a more detailed medical history of the non-hospitalized group could not be gathered. In addition, recurrences or infections diagnosed for the first time cannot be identified. Each patients’ age and gender were also considered in the research.

### Inclusion and exclusion criteria

Among the inclusion criteria, we identified a positive urine test with the presence of at least 105 CFU/mL, a single microbial agent identified on culture, age of at least 18 years, and male gender. Patients with bacteriuria lower than 105 CFU/mL, female patients, patients with multiple bacteria identified on culture, and cases in which the same urine test results were identified in the same patient at two different evaluations were excluded from the present study.

### Antibiotic policy

The European guidelines [12], revised annually, were mostly used to determine the prudent policy for the use of antibiotics in treating patients with UTIs. The patients were administered the minimal therapeutic regimen in accordance with the specified guidelines for various disorders, whenever feasible, based on the results of urine samples. The variation in infection sites and the degree of severity necessitate specific approaches to antibiotic administration. Based on the current European recommendations, it is uncommon for males to be diagnosed with cystitis, and it is regarded as a complicated urinary tract infection.

### Sample collection, bacterial isolation, and antibiotic test

Urine samples were incubated for 24 hours after being inoculated with a sterile disposable loop on standard inoculation plates. Urine samples obtained from a sterile container were used to culture the microorganisms on Columbia sheep agar and lactose agar. In additional instances, *Staphylococcus spp*. was cultivated on Chapman medium. Significant microbial growth, colony morphology (e.g., color, size, and texture), and characteristics were observed on a culture medium. Bacterial concentrations greater than 10^5^ CFU/mL of no more than two microorganism species were deemed significant. The bacteria were identified using colony morphology, Gram stain, and numerous standard biochemical assays (lactose fermentation, catalase, oxidase, indole, methyl red, etc.). The zones of antibiotic inhibition were interpreted in accordance with the Clinical and Laboratory Standards Institute (CLSI) guidelines for Antimicrobial Susceptibility Testing (AST), which was conducted using the Kirby–Bauer disk diffusion technique [18]. Previous publications have discussed bacterial culture, identification, and antibiotic susceptibility assays that have been implemented [3,15,19].

### Statistical analysis

The obtained data were indexed on an Excel file using Microsoft Excel (version 2016, Microsoft Corporation, Redmond, WA, United States). The descriptive statistical analysis was made using Python 3.8.9 (Python Software Foundation) version for macOS using the libraries jupyterlab 3.2.4 and pandas 1.3.4.

## Results

The present study documented 733 positive urine tests among male patients, which were separated from the beginning into Gram-negative (579 cases; 78.99%) and Gram-positive (154 cases; 21.01%; Table 1). Among the Gram-negative group, *E. coli* was the most prevalent in 41.45% of the cases, closely followed by *Klebsiella spp*. in 34.36%. *Enteroccocus spp*. was, as it was expected, the most isolated bacteria among the Gram-positive group, but only encountered 17.6% in the overall analyzed population.

**Table 1:** Bacterial prevalence

	Bacteria	n	%.
**Gram-negative**	*E. coli*	240	32.74
	*Klebsiella spp.*	199	27.15
	*Proteus spp.*	96	13.1
	*Pseudomonas spp.*	44	6.0
**Gram-positive**	*Enterococcus spp.*	129	17.6
	*Staphylococcus spp.*	25	3.41
**Total**		733	100

An additional important aspect of the current investigation would involve examining the frequency of bacteria detected within different age cohorts. According to the data presented in Table 2, a significant percentage of male patients diagnosed with urinary tract infections were older than 55 years, irrespective of the specific uropathogen under investigation. The graph illustrates a gradual rise in the prevalence of UTIs caused by *E. coli* and *Klebsiella spp*. until the age of 55. However, after this threshold, there is a sharp increase, with over 80% of analyzed patients diagnosed with UTIs.

**Table 2:** Bacterial prevalence according to age distribution

Bacteria	<40 years		41-55 years		>55 years	
	*n*	%.	*n*	%	*n*	%.
**Gram-negative**	17	2.93	57	9.84	505	87.21
*E. coli*	6	2.4	25	10.42	209	87.08
*Klebsiella spp.*	2	1.01	24	12.06	173	86.93
*Proteus spp.*	9	9.38	6	6.25	81	84.38
*Pseudomonas spp.*	0	0	2	4.55	42	95.45
**Gram-positive**	0	0	9	5.84	145	94.15
*Enterococcus spp.*	0	0	6	4.65	123	95.35
*Staphylococcus spp.*	0	0	3	12	22	88
**Total**	17	2.31	66	9	650	88.67

The primary objective of this study was to assess the prevalence of antibiotic resistance among uropathogens in male patients across different classes of antibiotics. Consequently, the resistance rates of the bacteria under investigation were then categorized into the primary groups of Gram-negative and Gram-positive bacteria (Tables 3 and 4).

**Table 3:** Gram-negative susceptibility and resistance patterns

Antibiotic Tested	E.coli				Klebsiella spp.				Pseudomonas spp.				Proteus spp.			
	R		S		R		S		R		S		R		S	
	*n*	%.	*n*	%.	*n*	%.	*n*	%.	*n*	%.	*n*	%.	*n*	%.	*n*	%.
**Amoxicillin-Clavulanic acid**	78	35.78	140	64.22	110	57.29	82	42.71	7	87.5	1	12.5	28	31.46	61	68.54
**Ampicillin**	0	0.0	1	100.0	3	42.86	4	57.14	0		0		0		0	
**Aztreonam**	28	12.44	197	87.56	77	40.74	112	59.26	19	46.34	22	53.66	10	10.99	81	89.01
**Trimethoprim-Sulfamethoxa zole**	9	47.37	10	52.63	11	50.0	11	50.0	0		0		4	66.67	2	33.33
**Ceftazidime**	23	10.0	207	90.0	74	38.95	116	61.05	15	35.71	27	64.29	11	11.83	82	88.17
**Fosfomycin**	3	1.44	205	98.56	2	20.0	8	80.0	0	0.0	3	100.0	0	0.0	7	100.0
**Imipenem**	1	0.43	233	99.57	25	13.59	159	86.41	21	50.0	21	50.0	11	11.7	83	88.3
**Levofloxacin**	84	36.52	146	63.48	76	40.0	114	60.0	20	51.28	19	48.72	35	38.89	55	61.11
**Linezolid**	1	50.0	1	50.0	0	0.0	4	100.0	0		0		0		0	
**Meropenem**	1	0.46	218	99.54	24	12.9	162	87.1	21	47.73	23	52.27	3	3.19	91	96.81
**Nitrofurantoin**	10	6.45	145	93.55	45	56.96	34	43.04	1	100.0	0	0.0	3	25.0	9	75.0
**Penicillin**	0	0.0	4	100.0	0	0.0	4	100.0	0		0		0	0.0	3	100.0
**Vancomycin**	0	0.0	4	100.0	0	0.0	4	100.0	0		0		0	0.0	3	100.0

R, Resistance; S, Susceptibility; n, Number; %, Percentage

**Table 4:** Gram-positive susceptibility and resistance patterns

Antibiotics Tested	Enterococcus spp.				Staphylococcus spp.			
	R		S		R		S	
	*n*	%.	*n*	%.	*n*	%.	*n*	%
**Amoxicillin-Clavulanic acid**	5	83.33	1	16.67	0		0	
**Ampicillin**	8	6.67	112	93.33	0	0.0	1	100.0
**Aztreonam**	2	66.67	1	33.33	0		0	
**Trimethoprim-Sulfamethoxazole**	0	0.0	3	100.0	8	33.33	16	66.67
**Ceftazidime**	2	66.67	1	33.33	0		0	
**Fosfomycin**	4	3.6	107	96.4	0	0.0	1	100.0
**Imipenem**	1	33.33	2	66.67	0		0	
**Levofloxacin**	55	56.7	42	43.3	9	52.94	8	47.06
**Linezolid**	1	0.9	110	99.1	1	4.0	24	96.0
**Meropenem**	0	0.0	3	100.0	0		0	
**Nitrofurantoin**	2	1.72	114	98.28	2	10.53	17	89.47
**Penicillin**	24	19.67	98	80.33	13	68.42	6	31.58
**Vancomycin**	0	0.0	125	100.0	0	0.0	1	100.0

R, Resistance; S, Susceptibility; n, Number; %, Percentage

In the case of *E. coli*, the highest rates of resistance in a significant number of patients were observed for Levofloxacin (36.52%) and Amoxicillin-Clavulanic acid (35.78%). They were followed by Aztreonam (12.44%) and Ceftazidime (10.0%). Preserved resistance rates were observed in Fosfomycin, Nitrofurantoin, and Carbapenems. In the case of the second most common Gram-negative bacterium, *Klebsiella spp*. showed even more alarming rates with the studied antibiotics. Thus, the resistance rate exceeded 50% in the case of Amoxicillin-Clavulanic acid (57.29%) and Nitrofurantoin (56.96%). More than 40% of patients with this bacterium showed resistance to Ampicillin (42.86%), Aztreonam (40.74%), and Levofloxacin (40.0%). Ceftazidime (38.95%) was also very close to those already discussed. For carbapenems, the numbers also did not look very promising. Imipenem (13.59%) and Meropenem (12.9%) already exceeded the 10% resistance rate.

For *Proteus spp*. the highest resistance rate revealed in the present study was detected for Levofloxacin (38.89%), followed by Amoxicillin-Clavulanic acid (31.46%). Over 10% pattern was registered for Ceftazidime (11.83%), Imipenem (11.7%), and Aztreonam (10.99%).

*Pseudomonas spp.*, with the lowest prevalence among Gram-negative bacteria, exhibited alarmingly high resistance rates to Levofloxacin (51.28%), Imipenem (50.0%), Meropenem (47.73%), Aztreonam (46.34%), and Ceftazidime (35.71%).

For Gram-positive bacteria, significant results in terms of the number of analyzed patients were observed in the case of *Enterococcus spp*.

Important results regarding the antibiotic susceptibility pattern were reflected by resistance to Levofloxacin (56.7%) and Penicillin (19.67%). Preserved rates were observed in the case of Ampicillin (6.67%) and Vancomycin (0%). Fosfomycin (3.6%) also revealed good prospects in terms of resistance. For Carbapenems, we considered the number of the included cases to be small to make a valid presumption.

This study included in the analysis the overall rates established for all age groups established for both the Gram-negative and Gram-positive bacteria groups.

In the case of Gram-negative uropathogens, increased resistance patterns of over 30% were observed in the case of Amoxicillin-Clavulanic acid (43.98%), and Levofloxacin (39.16%). Aztreonam (24.54%), Nitrofurantoin (23.89%), and Ceftazidime (22.16%) have been recorded with resistance rates between 20% and 30%. An important aspect in the case of this group of bacteria can be observed in the case of Imipenem (10.47%) and Meropenem (9.02%), where the resistance was around 10%. Encouraging sensitivity was established for Fosfomycin (97.81%), which might prove its utility for male patients in intravenous administration.

The other studied group in the overall analysis, the Gram-positive, revealed increased resistance for Levofloxacin (56.14%) and Penicillin (26.24%) (Table 5). Preserved sensitivity to active antibiotics was observed for Ampicillin (93.39%) and Vancomycin (0.0%). Fosfomycin (96.43%) also showed a preserved sensibility in this case.

**Table 5:** Overall Gram-negative and Gram-positive antibiotic susceptibility and resistance patterns

Antibiotics Tested	Gram-negative				Gram-positive				Total			
	R		S		R		S		R		S	
	*n*	%.	*n*	%.	*n*	%.	*n*	%.	*n*	%.	*n*	%.
**Amoxicillin-Clavulanic ac**.	223	43.98	284	56.02	5	83.33	1	16.67	228	44.44	285	55.56
**Ampicillin**	3	37.5	5	62.5	8	6.61	113	93.39	11	8.53	118	91.47
**Aztreonam**	134	24.54	412	75.46	2	66.67	1	33.33	136	24.77	413	75.23
**Trimethoprim-Sulfamethoxazole**	24	51.06	23	48.94	8	29.63	19	70.37	32	43.24	42	56.76
**Ceftazidime**	123	22.16	432	77.84	2	66.67	1	33.33	125	22.4	433	77.6
**Fosfomycin**	5	2.19	223	97.81	4	3.57	108	96.43	9	2.65	331	97.35
**Imipenem**	58	10.47	496	89.53	1	33.33	2	66.67	59	10.59	498	89.41
**Levofloxacin**	215	39.16	334	60.84	64	56.14	50	43.86	279	42.08	384	57.92
**Linezolid**	1	16.67	5	83.33	2	1.47	134	98.53	3	2.11	139	97.89
**Meropenem**	49	9.02	494	90.98	0	0.0	3	100.0	49	8.97	497	91.03
**Nitrofurantoin**	59	23.89	188	76.11	4	2.96	131	97.04	63	16.49	319	83.51
**Penicillin**	0	0.0	11	100.0	37	26.24	104	73.76	37	24.34	115	75.66
**Vancomycin**	0	0.0	11	100.0	0	0.0	126	100.0	0	0.0	137	100.0

R, Resistance; S, Susceptibility; n, Number; %, Percentage

An overall analysis for all the studied classes was done for different age groups. In the case of people younger than 40 years old, a lower rate of resistance to the tested antimicrobials could be observed, but the group includes a reduced number of patients. The pattern of evolution of resistance increases following the evolution in age of the male population, as can be seen in Table 6. Patients aged between 41 and 55 years showed lower resistance to the types of antibiotics studied than older patients. Analyzing two of the antibiotics that presented alarming rates in the group of Gram-negative bacteria, Levofloxacin and Amoxicillin-Clavulanic acid, the evolution among different ages, highlighted the following. For Levofloxacin 26.67% in <40 years, 24.19% between 41-55 years, and 44.37% over 55 years old. The overall result for this antibiotic maintained over 40%.

**Table 6:** Antibiotic susceptibility and resistance patterns according to age distribution

Antibiotics Tested	< 40 years				41–55 years				>55 years				Total			
	R		S		R		S		R		S		R		S	
	*n*	%.	*n*	%.	*n*	%.	*n*	%.	*n*	%.	*n*	%.	*n*	%.	*n*	%.
**Amoxicillin-Clavulanic ac**.	6	37.5	10	62.5	26	50.0	26	50.0	196	44.04	249	55.96	228	44.44	285	55.56
**Ampicillin**	0		0		4	44.44	5	55.56	7	5.83	113	94.17	11	8.53	118	91.47
**Aztreonam**	0	0.0	15	100.0	8	14.29	48	85.71	128	26.78	350	73.22	136	24.77	413	75.23
**Trimethoprim-Sulfamethoxa zole**	0		0		4	100.0	0	0.0	28	40.0	42	60.00	32	43.24	42	56.76
**Ceftazidime**	0	0.0	17	100.0	12	21.43	44	78.57	113	23.3	372	76.7	125	22.4	433	77.6
**Fosfomycin**	0	0.0	8	100.0	0	0.0	30	100.0	9	2.98	293	97.02	9	2.65	331	97.35
**Imipenem**	0	0.0	17	100.0	5	8.77	52	91.23	54	11.18	429	88.82	59	10.59	498	89.41
**Levofloxacin**	4	26.67	11	73.33	15	24.19	47	75.81	260	44.37	326	55.63	279	42.08	384	57.92
**Linezolid**	0		0		0	0.0	8	100.0	3	2.24	131	97.76	3	2.11	139	97.89
**Meropenem**	0	0.0	17	100.0	5	9.26	49	90.74	44	9.26	431	90.74	49	8.97	497	91.03
**Nitrofurantoin**	1	20.0	4	80.0	7	25.0	21	75.0	55	15.76	294	84.24	63	16.49	319	83.51
**Penicillin**	0		0		4	44.44	5	55.56	33	23.08	110	76.92	37	24.34	115	75.66
**Vancomycin**	0		0		0	0.0	6	100.0	0	0.0	131	100.0	0	0.0	137	100.0

R, Resistance; S, Susceptibility; n, Number; %, Percentage

In the case of Amoxicillin-Clavulanic ac., the evolution pattern revealed 37.5% in less than 40 years, 50% between 41-55, and 44.04% over 55 years old. The same as in Levofloxacin, the overall result exceeded 40%.

Other antibiotics also showed an increased resistance trend of evolution between the 41-55 years old and over 55 years old.

For instance, Aztreonam revealed a 14.29% resistance rate between 41-55, compared to 26.78% over 55 years.

In contrast to these already discussed, Ceftazidime, Imipenem, and Meropenem showed a linear evolution between 41-55 years and over 55 years. Although Ceftazidime expressed over 20% resistance in both 41-55 and over 55 groups, its evolution trend did not seem to suffer modification regarding increasing age. Imipenem and Meropenem evolution were also stable at around 10% in the studied age groups.

Two antibiotics showed a regression in their resistance evolution regarding age: Nitrofurantoin from 25.0% to 15.76% and Penicillin from 44.44% to 23.08%, but in the case of Penicillin, the 41**–**55 years old included a very small number of patients.

According to this observational and descriptive analysis, Fosfomycin was the only antibiotic tested that registered an encouraging sensibility pattern among all ages, from 0.0% to a maximum of 2.98%. Vancomycin also registered the same values, but it was only evaluated for Gram-positive cases.

A very good resistance profile was observed for Linezolid (2.24%), but, unfortunately, a significant number of cases were only registered in the over-55-year-old group.

## Discussion

The increasing prevalence of antibiotic resistance among uropathogens is a significant public health concern, which has gathered heightened attention in recent times. This issue is widely recognized as one of the foremost risk factors impacting the safety and well-being of the global population. The escalating utilization of antibiotics on a global scale by healthcare systems, the routine use of initial-line antibiotics for uncomplicated urinary tract infections, and the unrestricted availability of these drug classes in numerous developing nations has resulted in a significant surge in resistance rates to the most prevalent antibiotic classes in recent years [20].

UTIs exhibit a greater prevalence in the female population as compared to males, with an overall occurrence rate exceeding 80% in females. Furthermore, the likelihood of recurrence is higher in women. Research indicates that nearly 30% of women who have experienced a UTI will encounter another episode within 6 months following the initial occurrence. Additionally, approximately 48% of recurrent UTIs will manifest within one year [21].

Considering that most of the data in the literature are based on studies influenced by the prevalence and bacterial resistance observed in the female sex, all the treatment recommendations suggested by the current guidelines are based on this information [12].

Considering that the pathological factors correlated with the presence of urinary infections in the male sex, and that their prevalence is different from that found in the female sex, it is useful to know the most accurate data in order to be able to approach the most suitable therapeutic behavior.

### Bacterial prevalence and its evolution regarding age

The current study highlights *E. coli* as the most widespread bacterium among the male population studied. The second most prevalent bacteria were *Klebsiella spp*., followed by *Enterococcus spp*. The overall prevalence for *E. coli* (32.74%), although lower than in other studies performed among women, reflects this. Compared to the women, the reported data reveal over 60% prevalence for this uropathogen in several studies from Europe: France [22], Switzerland [23], and Portugal [24]. Also, increased values for *E. coli* prevalence were reported outside Europe in countries like Morocco [25], Pakistan [26], South Korea [27], and North America [28]. Compared to all worldwide data, this study reflects the almost half percentage prevalence of *E. coli* in the male population. On the other hand, when compared to similar studies including male patients, the present results are similar to those reported by studies from the Netherlands [29].

In the case of the second most common bacteria found in the population analyzed by this study, *Klebsiella spp*. (27.15%), the prevalence data demonstrate a much higher percentage than the data reported for women. A recent study from Hungary [30] revealed a 13.4% prevalence for *Klebsiella spp*. Also, other data from Turkey [31] present a prevalence of 11.2% for this analyzed uropathogen in the female population. This difference between sexes may be caused by different pathologies, which may require instrumentation (for example, urethral catheterization for acute urinary retention). For *Enterococcus spp*., there were no established correlations with recent data found in the literature because the reported prevalence varies a lot in several studies from 2.8% in Pakistan [32] to 21.3 in Nepal [33].

The present study reveals preserved data for bacterial prevalence evolution when compared to the results obtained on the Romanian male population in a multicentric study that analyzed patients from 1 September 2018 to 31 January 2019 [15]. These data may vary across the European continent as several countries, like Norway and Italy, reported different results [34,35].

The prevalence of asymptomatic bacteriuria is low in young male patients, but it significantly rises to 10% in men aged 60 years and older, and up to 40% among patients residing in long-term care institutions. The occurrence of UTIs in men is significantly influenced by the process of aging, as we have discovered a clear and direct correlation with advancing age [36]. In the present study, the UTI prevalence surpasses 80% after 55 years old. Compared to the opposite sex, urinary tract infections appear to have a different distribution pattern, considering that most studies show a uniform distribution during a lifetime [37]. The previous study that the same team conducted on the Romanian male population also revealed an increased incidence in the elderly [15].

### Resistance pattern in Gram-negative

The primary objective of this study was to evaluate the prevalence and rates of antibiotic resistance among uropathogens in male patients across different classes of antibiotics and to observe certain patterns in order to implement targeted measures.

This study highlighted the overall results with alarming values of resistance rate for Amoxicillin-Clavulanic acid and Levofloxacin in Gram-negative bacteria. These aspects are mostly reflected by all the actual research for both sexes [38].

The data from this study support the current evolution trend of *E. coli* resistance to Levofloxacin and Amoxicillin-Clavulanic acid of over 30%, similar to data evaluated for female sex, which already underlined the necessity of introducing some measures in administering empirical treatment because of Levofloxacin resistance [39]. Similar results revealed by studies conducted in Spain were also obtained in male cases in 2019 [40]. Research data indicate that the resistance of *E. coli* to Levofloxacin is part of a broader trend observed across various regions, driven by factors like the overuse and misuse of antibiotics [41,42]. This has led to a growing body of evidence suggesting that empirical treatment protocols need urgent evaluation to combat the rising tide of antimicrobial resistance [43]. The issue is particularly pressing given that Levofloxacin has been a cornerstone in treating UTIs, where often *E. coli* is the primary pathogen [44].

In light of these findings, experts advocate for more stringent antibiotic stewardship programs, which include better diagnostics to ensure appropriate use of antibiotics, as well as ongoing surveillance of resistance patterns to guide treatment decisions [45]. Additionally, there is a call for the development of new antibiotics and alternative therapies to address the gap left by the declining efficacy of existing drugs [46].

The growing prevalence of antibiotic resistance in *E. coli* underscores the importance of implementing targeted measures to optimize antibiotic use and mitigate treatment failures [47].

As resistance to Levofloxacin becomes more pronounced, clinicians must consider local resistance patterns to ensure effective management of infections and safeguard patient outcomes.

The second most prevalent bacteria analyzed, *Klebsiella spp*., showed over 50% resistance to Amoxicillin-Clavulanic acid and Nitrofurantoin. Ampicillin, Levofloxacin, and Ceftazidime also presented over 30% resistance rates. Results from the male population in 2020 showed 65% resistance rate for Amoxicillin-Clavulanic acid, but a lower rate for Nitrofurantoin of 23%, with an important 45% rate for Levofloxacin [48]. A study that aimed to assess antibiotic resistance in the female population before and after the COVID-19 pandemic showed an increase in resistance for quinolones in these patients from 16.87% to 35.51% [49]. Also, other studies that evaluated female populations showed that *Klebsiella spp*. was the second most common Gram-negative UTIs [49,50]. Interestingly enough, we observed an alarmingly high rate of resistance to Carbapenems, over 10%. Other studies that evaluated carbapenem resistance in female populations showed lower rates, less than 5% [50].

For *Proteus spp*. the highest resistance rate revealed in the present study was detected for Levofloxacin, almost 40%, followed by Amoxicillin-Clavulanic acid, over 30%. Other studies showed a lower resistance rate, of 24% for Levofloxacin, also in the male population [48]. Resistance around 10% was registered for Ceftazidime and Imipenem. Results are similar to previous data in the female population observed over a period of time [50], in the same locoregional situation. The same resistance pattern in different populations but from the same region is an interesting observation that needs further analysis to determine common factors that can explain a possible association. At the same time, another study that evaluated resistance patterns in males also showed a 15% [48].

*Pseudomonas spp*. had the lowest prevalence among the Gram-negative bacteria, but with the highest resistance rate for most of the antibiotics evaluated, numbers somewhat expected for Levofloxacin, but rates of roughly 50% for carbapenems also. Slightly lower rates were observed for Ceftazidime. Our results for *Pseudomonas spp*. seem to follow the same rates of prevalence and resistance in other populations over different periods of time [50]. During the COVID-19 pandemic, a significant increase in resistance was observed for Pseudomonas, from 30% to over 70% [49], while maintaining the same low prevalence.

### Resistance pattern in Gram-positive

The most common Gram-positive bacteria was *Enterococcus spp.*, as evident in many other studies [50,49]. The resistance rate to Levofloxacin is higher than in previous studies, in the male population [48] or the female population [49], from the same region, but also higher than the 30% rate in a study from Hungary [51]. The resistance rates for Penicillin were similar to other published rates [49], in female populations, but when compared to a similar study that also evaluated male patients, we found lower rates (26,24% compared to 33.52-66%) [50].

A regression of resistance was observed in the case of Nitrofurantoin and Penicillin, results that are different from the 3-year evaluation of a female population that showed similar rates for Penicillin and an upward trend for Nitrofurantoin [50]. Still, the Nitrofurantoin resistance rates are low, this antibiotic showing high sensitivity, results that are similar to most of the data from the literature [52], making this antibiotic an option in certain cases.

The evaluation of resistance evolution pre and post Covid pandemic showed significant increases to most of the antibiotics evaluated, no regression being observed [49]. At the same time, the trend of resistance evaluated from 2018 to 2022, showed also a decrease in resistance rates for carbapenems and Ceftazidime [50].

Fosfomycin was the only antibiotic with encouraging sensitivity, and Vancomycin showed similar values for Gram-positive cases, results that are similar to previously published data, also on the male population, with high sensitivity for these two antibiotics, over 87%, almost 94% in the case of Fosfomycin [48].

The highest sensitivity rate was observed for Linezolid, results that are similar to existing data [48]. High sensitivity to Linezolid is evident in other studies also [53].

The values regarding *Staphylococcus spp*. were similar to previous published data, with Penicillin having a resistance rate over 60% [48]. At the same time, the Levofloxacin rate found was higher (59% versus 36%) [48]. Taking into consideration that the assessed populations are similar, other factors might be accountable [54].

### Comparison

Our study supports the trend that male patients had severe antibiotic resistance to several antibiotics compared to females. At the same time, the comparison between men and women regarding antibiotic resistance show distinct patterns and variations in most of the studies in the literature.

Women show higher susceptibility to infections but lower resistance rates to some antibiotics. *Escherichia coli*, the most frequent pathogen in women, presented 35.98% resistance to levofloxacin and 28.62% to amoxicillin-clavulanic acid. These rates were slightly lower than those in male patients, respectively. *Klebsiella spp*. showed similar trends, with higher resistance rates in men compared to women across several antibiotics, notably Levofloxacin [15], but also a higher resistance to Amoxicillin-Clavulanic acid [55]. Resistance to Levofloxacin in Escherichia coli also displayed a male-dominated resistance pattern, with 36.52 % in men versus 28.62% in women.

These numbers illustrate that men generally have higher resistance rates than women across multiple antibiotics, especially for *E. coli* and *Klebsiella spp*.

## Conclusion

Overall, the male population shows consistently high resistance rates to several antibiotics, possibly due to the more complicated nature of their infections. This difference highlights the need for tailored therapeutic approaches based on gender-specific resistance patterns.
